# Atrial septal defect: hemodynamic changes before and after closure assessed with magnetic resonance exercise imaging

**DOI:** 10.1186/1532-429X-13-S1-P185

**Published:** 2011-02-02

**Authors:** Pia K Schuler, Philipp Lurz, Vivek Muthurangu, Rod Jones, Andrew M Taylor

**Affiliations:** 1 Great Ormond Street Hospital, London, UK; 2 Herzzentrum, Leipzig, Germany

## Introduction

ASDs are among the most common congenital cardiac anomalies seen in the adult population and closure leads to a significant improvement in cardiopulmonary function.

## Purpose

This study sought to identify the shunt dynamics during exercise and the hemodynamic mechanisms explaining the functional improvement after atrial septal defect (ASD) closure.

## Methods

10 patients (age 34.6 ± 12 years) meeting the criteria for defect closure (large defect, 27.5 ± 6.5 mm, with evidence of right ventricular volume overload) were included. MRI was performed at rest and during supine exercise stress at the same intensity pre- and post- (9.3 ± 8.3 months) closure. Bi-ventricular volumes and function were assessed under free breathing and continuation of exercise using a radial *k-*t SENSE real-time sequence.

## Results (see table 1)

**Table 1:** 

	Rest		Δ REST vs. EXERCISE (Δ exercise change pre/post closure as %)	
	pre-ASD	post-ASD	pre-ASD	post-ASD

RV EDV, ml/m^2^	116.5 ± 21.03	87.7 ± 21.2*	-8.3 ± 0.02	-2 ± 2.3 (-76%)
RV ESV, ml/m^2^	61.9 ± 14.74	43.7 ± 15.1*	-10 ± 3.8†	-7.9 ± 1.9 (-21%)
RV SV, ml/m^2^	54.1 ± 17.4	43.9 ± 8.4	2 ± 3.9	6.1 ± 0.46† (+20%)
RV EF, %	46.4 ± 10.2	51.6 ± 8.7	5.4 ± 4.3	8.5 ± 4.2† (+57%)
RV CI [ml/min *m^2^]	4.33 ± 16.1	3.1 ± 0.4*	2.1 ± .34†	2.1 ± 1.3† (+0%)
LV EDV, ml/m^2^	52 ± 14.6	68.4 ± 16.9*	-0.3 ± 2.2	0.4 ± 1.4 (+233%)
LV ESV, ml/m^2^	16.7 ± 6.1	21.9 ± 9.1	-2.3 ± 1.4	-3.1 ± 1.4† (-35%)
LV.RV effective SV, ml/m^2^	35.1 ± 9.8	46.5 ± 9.9*	2.5 ± 0.7	3.4 ± 1.3 (+36%)
LV EF, %	68.4 ± 7.06	69 ± 7.6	5.3 ± 0.06	5.2 ± 3† (=2%)
CILV [ml/min *m^2^]	2.74 ± 0.6	3.3 ± 0.55*	1.6 ± 0.65†	1.9 ± 0.9† (+19%)
Heart rate, bpm	79.9 ± 0.62	72.8 ± 12.2*	36.5 ± 9.5†	33,8 ± 17.3† (+7%)

*indicates a significant difference (p<0.05) between parameters assessed pre-ASD closure and post-ASD-closure

†indicates a significant difference (p<0.05) between parameters assessed @ rest and @ exercise

Before the closure there was little change in the magnitude of the left-to-right shunt during supine exercise (Qp:Qs at rest 1.54, at peak exercise 1.49) with exercise-induced tachycardia reducing diastolic shunt flow.

After ASD closure there was an improvement in filling of the left ventricle (LV). This was achieved by abolishment of the left-to-right shunting and by unloading the right ventricle (RV). The RV EDV and ESV reduction lead to a better RV contractile reserve during exercise (improved Frank Starling mechanism) increasing RV SV, EF and CI during exercise and causing less paradoxical movement of the interventricular septum.

This improved RV and LV SV augmentation during exercise resulted in a heart rate reduction providing a higher functional reserve.

## Conclusions

ASD shunt decreases during exercise. ASD closure improves RV contractile reserve, LV filling and cardiopulmonary function. MRI is an excellent technique to assess shunt volumes and hemodynamic changes during exercise.

